# An enhanced deep deterministic policy gradient algorithm for intelligent control of robotic arms

**DOI:** 10.3389/fninf.2023.1096053

**Published:** 2023-01-23

**Authors:** Ruyi Dong, Junjie Du, Yanan Liu, Ali Asghar Heidari, Huiling Chen

**Affiliations:** ^1^College of Information and Control Engineering, Jilin Institute of Chemical Technology, Jilin, China; ^2^School of Surveying and Geospatial Engineering, College of Engineering, University of Tehran, Tehran, Iran; ^3^College of Computer Science and Artificial Intelligence, Wenzhou University, Wenzhou, China

**Keywords:** robotic arm, intelligent control, reward function, experience replay mechanism, deep deterministic policy gradient algorithm, artificial intelligence, machine learning

## Abstract

Aiming at the poor robustness and adaptability of traditional control methods for different situations, the deep deterministic policy gradient (DDPG) algorithm is improved by designing a hybrid function that includes different rewards superimposed on each other. In addition, the experience replay mechanism of DDPG is also improved by combining priority sampling and uniform sampling to accelerate the DDPG’s convergence. Finally, it is verified in the simulation environment that the improved DDPG algorithm can achieve accurate control of the robot arm motion. The experimental results show that the improved DDPG algorithm can converge in a shorter time, and the average success rate in the robotic arm end-reaching task is as high as 91.27%. Compared with the original DDPG algorithm, it has more robust environmental adaptability.

## 1. Introduction

Methodologies for control engineering have been suggested as a potential protocol for integrating self-adaptive characteristics into software products (Li D. et al., 2021; Meng et al., 2021; Zhang et al., 2021). Intelligent robotic applications have become more adapted with the development of artificial intelligence (AI) technology (Wang et al., 2022c,d; Zhao et al., 2022). Robots are widely used in modern manufacturing to complete repetitive and dangerous manufacturing tasks.

The main obstacle to their growth is the motion control of the robotic arm. In past research, traditional control methods such as adaptive control (Martín-Sánchez et al., 2012), fuzzy control (Precup and Hellendoorn, 2011), and robust control (Spong, 1992) were usually used. Generally, these control methods’ stability and accuracy are limited, making it challenging to meet the rapidly evolving industrial needs (Wang et al., 2022a,b; Xu et al., 2022). As a result, many researchers improved and optimized robotic arm control algorithms. Ren and Dinghui (2020) used an adaptive sliding mode control method based on a fixed-time perturbation observer to enable the robotic arm to track a trajectory precisely and suppress the system’s jitter efficiently.

Liang et al. (2019) adopted the Cartesian space decoupling control and the reference load feed-forward compensation method to perform force-position hybrid control of the robotic arm, which improved the response speed and steady accuracy of the robotic arm. Soltanpour and Khooban (2013) proposed an optimal fuzzy sliding mode controller for robot position tracking, which overcame the uncertainty in robotic arm position tracking. Lu et al. (2016) proposed a single flexible robotic arm tip control method based on a linear quadratic regulator (LQR) based on the Mamdani model. The adaptive change of the LQR controlled variable R by adding a fuzzy algorithm to the conventional LQR controlled to improve the adaptability of the control system. These classical control methods suit industrial production environments with a single structure, where the robotic arm follows a predetermined trajectory to complete the production task (Hu et al., 2021). Optimization methods, generally, bring new ideas to solve complex engineering problems (Dong et al., 2021; Sun et al., 2022). However, traditional control methods of robotic arms strongly depend on the environment model and have various drawbacks when operating robotic arms in unstructured scenarios. For example, when the environment changes, the mathematical model of the robot arm and the environment must be re-established, resulting in a low model reusability rate and increased labor costs.

Deep reinforcement learning (DRL) (Wang et al., 2020) can perceive complex inputs and learn independent policies, as reinforcement learning has shown great potential in solving many problems (Pan and Xu, 2016; Zhang et al., 2020; Yun et al., 2021; Kaur and Mittal, 2022; Raheb et al., 2022). DRL makes robotic arm control more smart, does not require accurate modeling of the environment, and can compensate for the shortcomings of traditional motion planning methods. Many researchers have recently investigated robotic arm control based on the DRL approach (Iriondo et al., 2019; Moreira et al., 2020; Jiang et al., 2021; Sekkat et al., 2021; Yang Y. et al., 2021). Finn et al. (2016) proposed an inverse optimal control algorithm based on the DRL approach. The algorithm can learn complex nonlinear cost representations of the need to manually complete feature engineering. It can be applied to high-dimensional systems with unknown dynamics to achieve motion control of robotic arms in realistic scenarios. Zhang et al. (2015) autonomously learned the ability to control each joint angle of the robotic arm based on the Deep Q Network (DQN) algorithm and using visual perception as a state input, thus achieving reaching the target of a three-degree-of-freedom robotic arm. Yang L. et al. (2021) proposed a DRL-based ball-striking method. The method has spin velocity estimation capability to predict the relative spin velocity of the ball and accurately hit the ball back. Lee et al. (2022) developed and tested a digital twin-driven DRL learning method for robots to prioritize tasks efficiently in dynamic construction environments. The deep deterministic policy gradient (DDPG) algorithm (Lillicrap et al., 2015) is another DRL algorithm for handling high-dimensional continuous action space tasks. The DDPG is a model-free control method based on modeling and is suitable for robotic arm motion control in dynamic environments. However, the original DDPG algorithm is time-consuming and inefficient in training models for handling control tasks in complex and variable environments. Moreover, if the reward function of DDPG is not designed properly, there is also a sparse reward problem (Jia et al., 2019), which prevents the robotic arm from learning the desired control strategy during the training process.

In recent years, DDPG algorithms have been applied in a wide range of scenarios, such as analyzing electricity market strategies (Liang Y. et al., 2020), battery-involved energy management (Wu et al., 2020; Wei et al., 2021), adaptive neuro-fuzzy proportional-integral-derivative (PID) control (Shi Q. et al., 2020), energy harvesting wireless communication management (Qiu et al., 2019), optimal control for batch processes (Yoo et al., 2021), target tracking in high-altitude scenes (Liang S. et al., 2020), energy consumption optimization for zinc electrowinning processes (Shi X. et al., 2020), performance optimization of car-following (Yan et al., 2021), etc. Li et al. (2019) proposed the minimax multi-agent (M3DDPG) algorithm, which uses multi-intelligence adversarial learning to solve the local optimum problem effectively. Li J. et al. (2021) proposed the multiple experience pool replay twin delayed DDPG (MEPR-TD3) algorithm to improve the training efficiency and action quality by playing back the strategy to multiple experience pools. Han et al. (2021) proposed a regular update deterministic policy gradient algorithm to improve data utilization in the replay buffer. Joshi et al. (2021) the twin-actor twin-delayed DDPG (TATD3) algorithm was proposed by incorporating twin-actor networks in the existing twin-delayed DDPG (TD3) algorithm for continuous control. Li X. et al. (2021) improved the sampling strategy of the experience pool by adopting a high-priority experience playback strategy and proposed the efficient multi-agent deep deterministic policy gradient (E-MADDPG) algorithm to improve the efficiency of path planning. Zhang et al. (2019) proposed asynchronous episodic DDPG (AE-DDPG) to achieve more efficient learning with less training time. Xie and Zhong (2020) proposed the semi-centralized deep deterministic policy gradient (SCDDPG) algorithm to design a two-level actor-critic structure and a reward function with a local and global structure based on the attributes of the bits of intelligence to improve the learning performance of the intelligence in a stochastic environment.

This paper proposes an improved DDPG algorithm for the intelligent control of a robotic arm to solve the problems mentioned above. It is based on a hybrid reward strategy and an improved experience replay mechanism.

(1) A hybrid reward function containing distance reward, sparse reward, direction reward, and area reward is designed by combining the robotic arm and the environment. It enables the robotic arm to learn the strategy of reaching the target object faster and more smoothly and improves the algorithm learning efficiency.

(2) The improved experience replay mechanism combines the advantages of preferential sampling and uniform sampling, reduces the correlation of sample data during training, and improves sample data quality. It is beneficial to improve the utilization rate of sample data and accelerate the speed of network training.

Experimental results show that the improved DDPG algorithm converges faster in training than the original DDPG algorithm. The final arrival of the robotic arm control model has a higher test success rate, resulting in better environmental adaptation capability and control accuracy of the robotic arm.

The structure of this paper is outlined as follows. Section “2. DDPG algorithm” briefly introduces the DDPG algorithm flow and how the Actor policy network and Critic evaluation network are updated. Section “3. Design of improved DDPG algorithm” details the improvements to the DDPG algorithm, including the design of the hybrid reward function and the experience replays mechanism. In Section “4. Analysis of experimental results,” the parameters of the reward function are determined experimentally, and the convergence speed and control performance of the original and improved algorithms are compared. Section “5. Conclusion and future works” summarizes and concludes the paper.

## 2. DDPG algorithm

**FIGURE 1:**
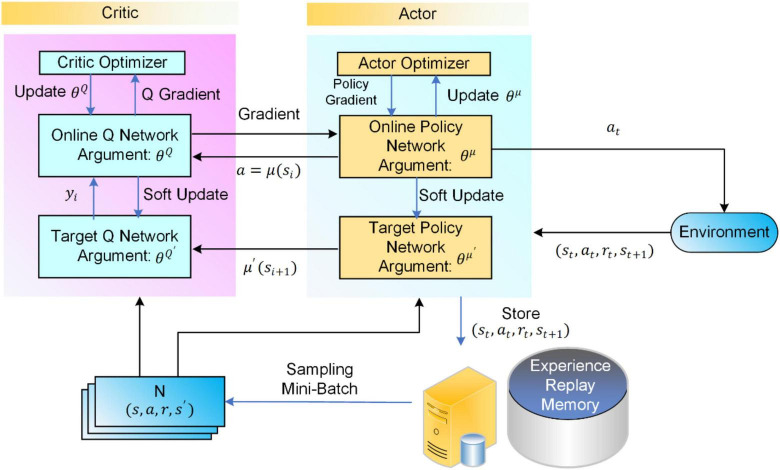
Flowchart of the deep deterministic policy gradient (DDPG) algorithm.

The Actor policy network of the DDPG algorithm is used to explore the environment and make action decisions; the Critic evaluation network is used to judge the merit of each action, which guides the direction of the gradient update of the Actor policy network. The framework structures and the specific process of the DDPG algorithm are shown in Figure 1.

Both Actor and Critic contain two neural networks with the same network structure, namely the online network and the target network. Random initialization of the online Q network *Q*(s,a|θ*^Q^*) and the online policy networked μ(s|θ^μ^) parameters of the θ*^Q^* and ^θμ^ and with the parameters ^θ^*Q*′^^←θ*^Q^* and ^θ^μ′^^←^θμ^ initialize their corresponding target networks ^*Q*′^ and ^μ′^. The action is selected according to the current policy *a_t_* as shown in Equ. 1.


(1) $$a_{t}=\mu \,\left(s_{t}|\theta^{\mu }\right)+N_{t}$$

where *a_t_* does the current policy select the executive action, *s_t_* is the current observed state, and *N_t_* is the exploration noise.

The DDPG algorithm decision processes to perform actions in the environment *a_t_* to get the return reward value *r_t_* and a newly observed state *s*_*t+1*_ and the sample data is (*s*_*t*_,*a*_*t*_,*r*_*t*_,*s*_*t* + 1_) are stored in the experience pool Replay Memory. The target Q value of the Critic is calculated as in Equ. 2. The Critic is updated by minimizing TD deviation as shown in Equ. 3.


(2) $$y_{i}=r_{i}+\gamma \,Q^{\text{’}}\,\left(s_{i+1},\mu^{\text{’}}\,\left(s_{i+1}|\theta^{\mu^{\text{’}}}\right)|\theta^{Q^{\text{’}}}\right)$$

(3) $$L=\frac{1}{N}\,\sum_{i}{\left(y_{i}-Q\,\left(s_{i},a_{i}|\theta^{Q}\right)\right)}^{2}$$

where γ is the attenuation factor.

The Actor policy network updates the network parameters based on the Policy Gradient, as shown in Equ. 4.


(4) $$\nabla_{\theta^{\mu }}J\approx \frac{1}{N}\,\sum_{i}\left({{\nabla_{a}Q\,\left(s,a|\theta^{Q}\right)|}_{s=s_{i},a=\mu \,\left(s_{i}\right)}\cdot \nabla_{\theta^{\mu }}\mu \,\left(s|\theta^{\mu }\right)|}_{s=s_{i}}\right)$$

where $\nabla_{\theta^{\mu }}\mu \,\left(*\right)$ is the Actor-network gradient and _∇*a*_⁡*Q*(*) is the Critic network gradient. $\nabla_{\theta^{\mu }}J$ this allows the Actor to continuously adjust its network parameters in the maximum direction available reward θ^μ^.

The Actor’s online network is updated in real-time based on the Critic’s online network as a guide, which is updated in real-time using its target network as a guide. Therefore, the parameters of the online network are up to date. In contrast, the target network parameters are delayed based on the online network parameters using soft updates, as shown in Equ. 5.


(5) $$\left\{ \begin{array}{c} \theta^{Q^{\text{’}}}\leftarrow \tau \,\theta^{Q}+\left(1-\tau \right)\,\theta^{Q^{\text{’}}}\\ \theta^{\mu^{\text{’}}}\leftarrow \tau \,\theta^{\mu }+\left(1-\tau \right)\,\theta^{\mu^{\text{’}}} \end{array}\right.$$

where ^θ*Q*^’^^ is the target Q network parameter, ^θμ’^ is the target policy network parameter, and τ is the soft update constant.

The DDPG algorithm is based on the Actor-Critic framework that constantly interacts with the environment to train its network iteratively. This dual-network mechanism using both the online network and the target network of parameter updates allows for more stable parameter updates and effectively improves the learning efficiency of the algorithm.

## 3. Design of improved DDPG algorithm

**FIGURE 2:**
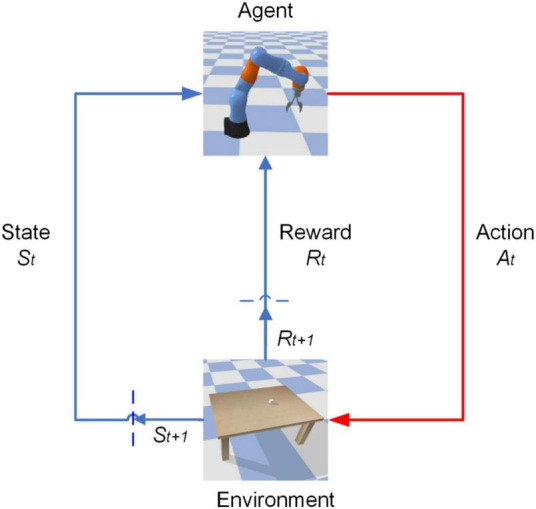
Robotic arms interacting with the environment.

The DDPG algorithm allows the robotic arm to interact with the environment continuously and get a large amount of sample data (*s*_*t*_,*a*_*t*_,*r*_*t*_,*s*_*t* + 1_) onto the update of the network parameters in the algorithm, as shown in Figure 2. With this iterative training, the control model of the robotic arm is finally output to achieve intelligent control of the robotic arm.

### 3.1. Design of hybrid reward functions

The DDPG algorithm can reduce redundant exploration and improves the training efficiency by setting the reward function of output the reward value of each action of the robotic arm so that the end of the arm can reach the target object quickly and stably under the training of the DDPG algorithm. However, in dynamic environments or complex tasks, the original DDPG algorithm suffers from the problem of reward sparsity. On the one hand, the strategy of intelligence is a random strategy at the beginning of training, and the acquisition of reward requires a series of complex operations. Hence, it is difficult for intelligence to obtain the reward under the initialized strategy, leading to training difficulties. On the other hand, sparse rewards are widely available in reinforcement learning tasks. For example, in the robotic arm arrival task, the robot arm successfully reaches the target through a series of complex positional controls before it can obtain a reward, and the failure of any intermediate step leads to the inability to obtain a reward. The sparse reward problem can lead to slow iteration and even complex convergence of the original DDPG algorithm. So this paper combines the robotic arm and the environment to design a hybrid reward function, including distance reward, sparse reward, direction reward, and area reward, so that the improved algorithm can learn the expected strategy and improve the learning efficiency.

#### 3.1.1. Distance reward function

It is difficult for the robotic arm to learn effectively in an unknown, exploratory environment with a large space. The distance from the robotic arm of the target object gives the corresponding reward. The smaller the distance, the larger the reward; vice versa, the smaller the reward. This way, it can shorten the exploration time and facilitate the algorithm’s learning to bring the robotic arm closer to the desired target object.

The distance between the end of the robotic arm and the target object *d*, is shown in Equ. 6.


(6) $$d=\sqrt{{\left(x_{a}-x_{o}\right)}^{2}+{\left(y_{\text{a}}-y_{o}\right)}^{2}+{\left(z_{a}-z_{o}\right)}^{2}}$$

where: the coordinates of the end of the robot arm are (*x*_*a*_,*y*_*a*_,*z*_*a*_) and the coordinates of the target object are (*x*_*o*_,*y*_*o*_,*z*_*o*_).

In order to make this reward function generalized in different environments, the distance *d* is transformed by the exponential calculation. The specific distance reward function is shown in Equ. 7.


(7) $$r_{1}=2\,\left(e^{-0.99\,d}-1\right)$$

where *r_1_* is the distance reward value; the smaller the distance value *d*, the larger the reward value. Regardless of *d* how large it is, the *r_1_* can be limited to the range of (−2,0), which facilitates setting other reward functions.

#### 3.1.2. Sparse reward function

Exploration of the robotic arm requires the identification of a learning task. In training, sparse rewards set a clear learning goal for the robotic arm. A large reward is given at the end of the robotic arm only when it reaches the target object, guiding the robotic arm to complete the desired task. Equ. 8 gives the sparse reward function.


(8) $$r_{2}=\left\{ \begin{array}{c} 0,\mathrm{target}\,\mathrm{is}\,\mathrm{not}\,\mathrm{reached}\\ R_{\text{sparse}},\mathrm{reaching}\,\mathrm{the}\,\mathrm{target} \end{array}\right.$$

where *r_2_* is the sparse reward value, and *R*_sparse_ is the reward value of reaching the target object at the end of the robotic arm.

#### 3.1.3. Directional reward function

The directional reward is designed for the movement of the robotic arm toward the position of the target object to reduce the blindness of the pre-exploration. If the end of the arm moves to the next step with a tendency to move closer to the target object, a positive reward is given, and vice versa for a negative reward. The direction of movement toward the robot arm of each step is gradually adjusted to the target object by smaller reward values. Equ. 9 gives the directional reward function.


(9) $$r_{3}=\left\{ \begin{array}{c} -R_{\text{direction}},d^{{}^{\prime }}>d\\ 0,d^{{}^{\prime }}=d\\ R_{\text{direction}},d^{{}^{\prime }}<d \end{array}\right.$$

where *r_3_* is the directional reward value that ^*d*′^ represents the distance between the end of the robotic arm and the target object in the next step, and −*R*_direction_ is the negative reward value for the movement of the robotic arm away from the target object, and *R*_direction_ is the reward value for the movement of the robot arm close to the target object.

#### 3.1.4. Regional reward function

It suffers low exploration efficiency, only relying on sparse rewards to complete pre-defined tasks. Area rewards can provide a practical exploration guide in the learning process of the robotic arm, reinforce the learning goal and improve the learning efficiency of the algorithm. When the end of the robot arm is about to reach the target, the corresponding reward value is given according to how close it is to the target; that is, the closer it is to the target, the greater the reward value with this enhanced reward *r_4_*. That allows the robotic arm to explore less environment and increases the chances of reaching the target object. Equ. 10 gives the area reward function.


(10) r4={Rzone1⁢ 0.05<d≤0.1Rzone2⁢ 0<d≤0.05

where *r_4_* is the regional award value, the *R*_zone1_ and *R*_zone2_ are the reward values set for the range of distances when the end of the robot arm approaches the target object.

The final hybrid reward functions are determined by superimposing the above four reward functions *r*, as shown in Equ. 11.


(11) $$r=r_{1}+r_{2}+r_{3}+r_{4}$$

### 3.2. Design of improved experience replays mechanism

The experience replay mechanism is used in the DDPG algorithm to reduce the relevance of data when training and exploring. The experience replayed pool stores a quaternion array (s_t_,a_t_,r_t_,s_t + 1_) that holds data samples from past robotic arm interactions with the environment. The experience replay mechanism enables the reuse of historical sample data and reduces wasted resources. The quality of the sample data has an important impact on network training. However, the original DDPG algorithm treats all the stored data onto the experience replay pool equally, and there is no prioritization between the data. The network parameters are trained in a uniform sampling manner.

**FIGURE 3:**
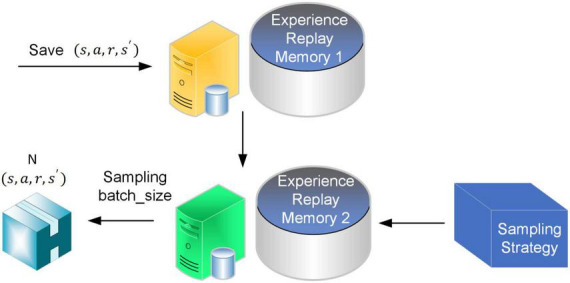
Improved experience pool.

To address the above matters, experience pool two is added behind the original experience pool, as shown in Figure 3. In experience pool 2, the original stored sample data is mixed with uniform sampling and priority sampling processing to ensure the quality of the selected sample data and improve its utilization.

**FIGURE 4:**
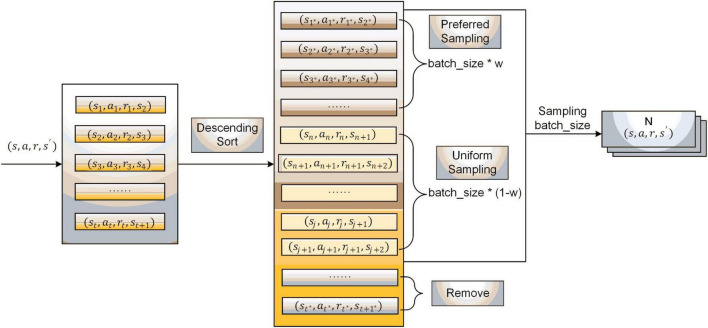
Experience pool data processing and sampling.

The sampling process of experience pool 2 is shown in Figure 4. First, the sample data are ranked in descending order according to the magnitude of their reward values; then, the data with small reward values at the bottom part of the experience pool are eliminated. After that, the data ranges between priority and uniform sampling and are divided according to a certain proportion. The sampling weight *w* is set, and *batch_size* * *w* and *batch_size* * (1−*w*) data are gained in the priority sampling set and uniform sampling set, respectively. Finally, the *batch_size* data are collected and used for the new batch of network training.

The improved sampling strategy combines the advantages of priority sampling and uniform sampling, preferentially selecting high-quality data with large reward values and eliminating some low-quality data onto small reference values, which is conducive to accelerating network training and promoting algorithm convergence.

### 3.3. Procedure of improved DDPG algorithm

**FIGURE 5:**
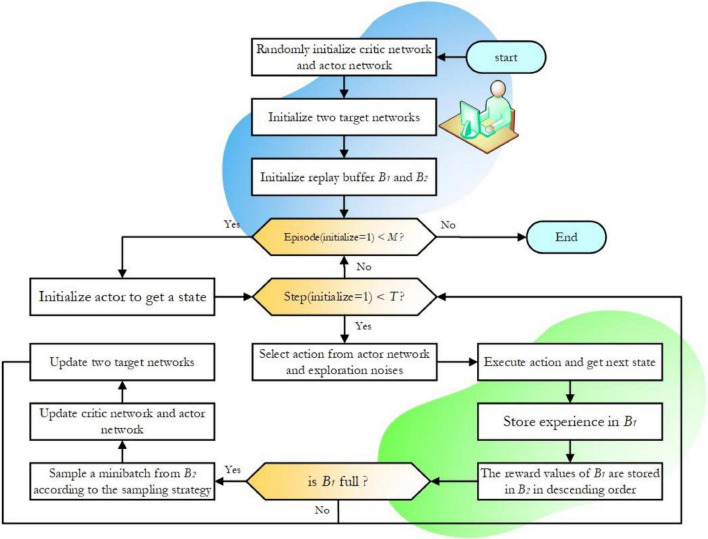
Flowchart of the improved deep deterministic policy gradient (DDPG) algorithm.

The pseudocode of the interaction procedure of the improved DDPG is given in Algorithm 1. The algorithm’s four network parameters and two experience pools need to be initialized at the beginning. Performing an action *a_t_* in each step will result in the corresponding reward value *r_t_* and the next state *s*_t+1_ according to the hybrid reward function. The sample data (s_t_,a_t_,r_t_,s_t + 1_) are deposited into the experience pool *B_1_*. Experience pool *B_2_* stores samples from *B_1_* after processing the data in Figure 4. When the experience pool *B_1_* is full, the minimum batch samples are obtained from the experience pool *B_2_* according to the improved sampling strategy, the sample data is used to update the network. The network parameters are updated iteratively in a single step during the training rounds. The algorithm flow chart of the improved DDPG is shown in Figure 5.

**Algorithm 1:** Improved deep deterministic policy gradient (DDPG) algorithm.

**1**	Randomly initialize critic network *Q*(s,a|θ*^Q^*) and actor-network μ(s|θ^μ^) with weights θ*^Q^* and θ^μ^
**2**	Initialize target network *Q*′ and μ′ with weights θ^*Q*′^←θ*^Q^*, θ^μ′^←θ^μ^
**3**	Initialize replay buffer *B_1_* and *B_2_*
**4**	**for** *episode* = 1, *M* **do**
**5**	Initialize a random process *N* for action exploration
**6**	Receive initial observation state *S_1_*
**7**	**for** *step* = 1, *T* **do**
**8**	Select action *a*_*t*_=μ(*s*_*t*_|θ^μ^) + *N*_*t*_ according to the current policy and exploration noise
**9**	Execute action *a_t_*, observe reward *r_t_* and next state *s*_*t+1*_
**10**	Store (*s*_*t*_,*a*_*t*_,*r*_*t*_,*s*_*t* + 1_) in the replay buffer *B_1_*
**11**	*B*_2_ is obtained by sorting *B^1^* in descending order of reward values and then removing the data with small reward values at the end
**12**	Set the weight *w*. *batch_size* * *w* and *batch_size* * (1−*w*) transitions (*s*_*i*_,*a*_*i*_,*r*_*i*_,*s*_*i* + 1_) are obtained in the proportionally divided priority sampling set and uniform sampling set of *B_2_*, respectively
**13**	Compute targets *y*_*i*_ = *r*_*i*_ + γ*Q*^’^(*s*_*i* + 1_,μ^’^(*s*_*i* + 1_|θ^μ’^)|θ^*Q*^’^^)
**14**	Update critic by minimizing the loss: $L=\frac{1}{N}\,\sum_{i}{\left(y_{i}-Q\,\left(s_{i},a_{i}|\theta^{Q}\right)\right)}^{2}$
**15**	Update the actor policy using the sampled policy gradient: $\nabla_{\theta^{\mu }}J\approx$ $\frac{1}{N}\,\sum_{i}\left({{\nabla_{a}Q\,\left(s,a|\theta^{Q}\right)|}_{s=s_{i},a=\mu \,\left(s_{i}\right)}\cdot \nabla_{\theta^{\mu }}\mu \,\left(s|\theta^{\mu }\right)|}_{s=s_{i}}\right)$
**16**	Update the target networks: θ^*Q*^’^^←τθ*^Q^* + (1−τ)θ^*Q*^’^^ θμ^’^←τθ^μ^ + (1−τ)θ^μ^’^^
**17**	**end for**
**18**	**end for**

## 4. Analysis of experimental results

To verify the effect of the improved DDPG algorithm, this paper builds a simulation environment based on the PyBullet platform with a Kuka seven-degree-of-freedom robotic arm to conduct experiments on reaching the target object position with the end of the robotic arm. Because of the physical constraints, hardware development no usually leads in enhanced performance in computational methods (Li, 2022; Li and Liu, 2022). Consequently, we have utilized a typical hardware with following details. The computer processor used for training is an Intel Xeon Gold 5218, the graphics card is an NVIDIA GeForce RTX 3090, and the algorithm is coded based on the TensorFlow framework on the Ubuntu operating system.

**FIGURE 6:**
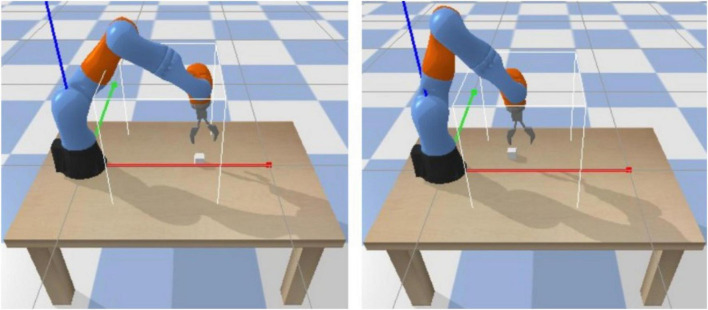
3D simulation environment for the robotic arm.

The robotic arm is placed on the operating platform, and its task is to reach objects within the limits of the solid white line. During the training process, the location where the target object appears is randomly generated at the initialization of each round, as shown in Figure 6.

### 4.1. Determination of reward parameters

**FIGURE 7:**
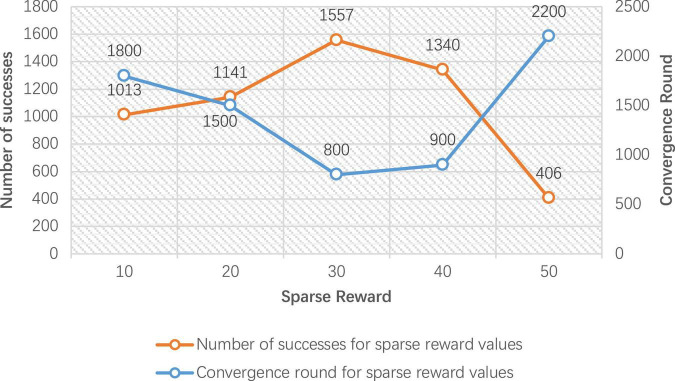
Number of successes and convergence rounds for 5,000 rounds of sparse reward training.

**FIGURE 8:**
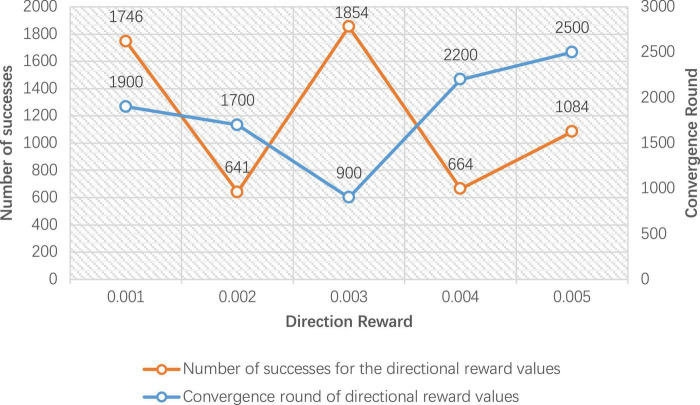
Number of successes and convergence rounds for 5,000 rounds of directional reward training.

**FIGURE 9:**
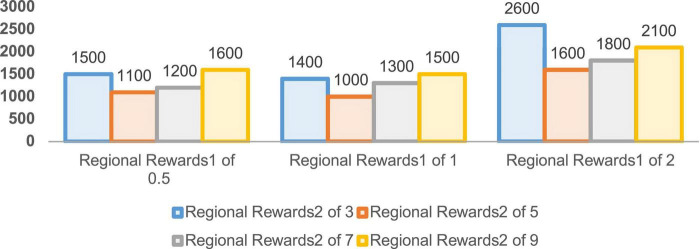
Convergence rounds for 5,000 rounds of regional reward training.

**FIGURE 10:**
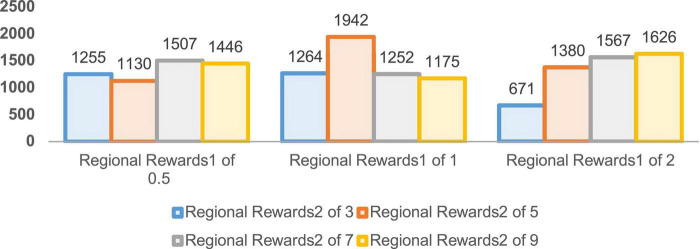
Number of successes in 5,000 rounds of regional reward training.

In order to verify the performance of the improved DDPG algorithm, it is necessary to determine the values of various reward parameters first, including sparse reward valuesR_sparse_, directional reward values R_direction_, and the regional reward values R_*zone1*_ and R_*zone2*_. Figures 7–10 are the result data for each set of 5,000 training rounds.

Figure 7 gives the convergence rounds and the number of successes of the improved DDPG algorithm for different sparse reward values *R*_sparse_. The convergence rounds are the number of rounds where the average step length stabilizes, and success counts are the number of completions where the end of the robot arm reaches the target object. It can be seen that the sparse reward value of 30 converges at 800 rounds, and the number of successes is 1,557, achieving the fastest convergence rounds and the highest number of successes. Therefore, in the improved DDPG algorithm, the sparse reward value *R*_sparse_ is set to 30.

Figure 8 gives the improved DDPG algorithm for the convergence rounds and the number of successes at different values of directional reward *R*_direction_. It can be seen that the directional reward value of 0.003 converges at 900 rounds, and the number of successes is 1,854, achieving the fastest convergence rounds and the highest number of successes. Therefore, in the improved DDPG algorithm, the directional reward value *R*_*direction*_ is set to 0.003.

The convergence rounds and success counts of the improved DDPG algorithm for different region reward values *R*_zone1_ and *R*_zone2_ are given in Figures 9, 10, respectively. It can be seen that the region reward value *R*_zone1_ with 1 and *R*_zone2_ with 5 converging at around 1,000 rounds with a success number of 1,942, reaching the fastest convergence round and the highest success number. Therefore, in the improved DDPG algorithm, the region reward values *R*_zone1_ and *R*_zone2_ are set to 1 and 5, respectively.

### 4.2. Comparison of convergence speed

**FIGURE 11:**
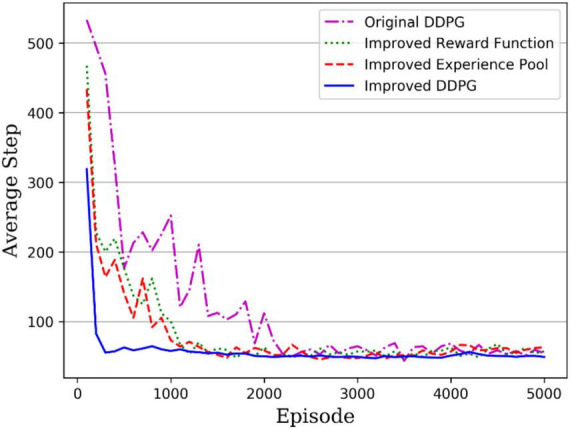
Average stride length per 100 rounds of training.

During the practical training, the maximum number of steps to complete the task per round was set to 1,000, and if the robotic arm could end within 1,000 steps, it was shown that the robotic arm reached task completion. To facilitate the comparison of training results, all experiments were set to the same number of rounds of 5,000. The average step length used per 100 rounds was recorded during the training. Figure 11 gives the convergence curves for the original and improved DDPG algorithms. The purple dotted line is the original DDPG algorithm; the green dotted line is the DDPG algorithm with improved reward, the red dashed line is the DDPG algorithm with improved experience pool, and the solid blue line is the DDPG algorithm with both improved reward and experience pool. The original DDPG algorithm converges at 2,200 rounds, the DDPG with improved reward function and DDPG with improved experience pool converge at around 1,100 and 1,400 rounds, respectively, and finally, the hybrid improved DDPG algorithm reaches convergence of around 200 rounds. Moreover, before convergence, the hybrid improved DDPG algorithm was below the convergence curve of the other DDPG algorithms and had higher training efficiency. Therefore, the hybrid improved DDPG algorithm accelerates the convergence speed compared with other DDPG algorithms.

**FIGURE 12:**
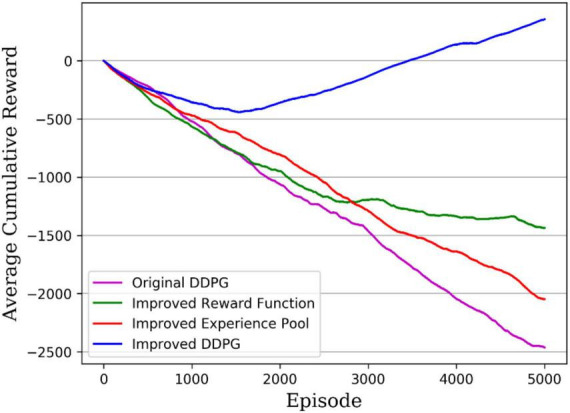
Average reward accrued from training.

Another measure of the algorithm performance is the average reward value per round accumulated during the algorithm’s training; the higher the cumulative reward value, the better the algorithm performance. Figure 12 gives the cumulative reward curves for different DDPG algorithms, with the original DDPG algorithm in solid purple, the DDPG algorithm with improved reward in solid green, the DDPG algorithm with improved experience pool in solid red, and the hybrid improved DDPG algorithm in solid blue. It can be seen that the hybrid improved DDPG algorithm received higher rewards, with the cumulative reward curve consistently above the other DDPG algorithm curves and significantly ahead of the other DDPG algorithms after 1,500 rounds. Therefore, the hybrid improved DDPG algorithm performs better than the other DDPG algorithms.

### 4.3. Comparison of control performance

**FIGURE 13:**
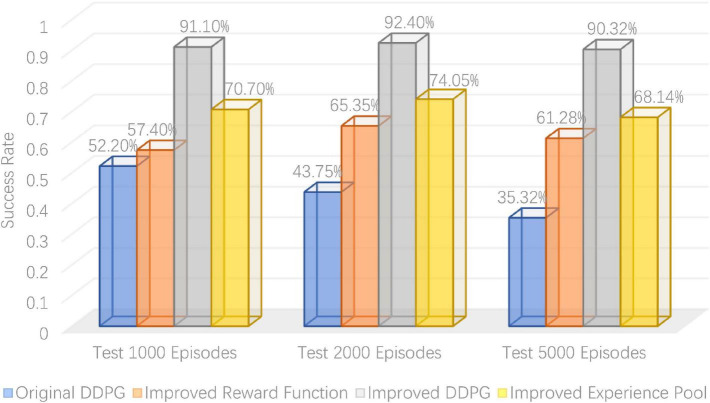
Visual comparison of test success rate.

To verify the robotic arm’s control performance, the DDPG algorithm’s success rate was tested before and after its improvement in the robotic arm reaching task. As shown in Table 1, the success rates of the robotic arm in reaching the target object at 1,000, 2,000, and 5,000 rounds were tested, with the bold ones being the highest success rates. It can be seen that the average success rate for the original DDPG algorithm, the DDPG algorithm with improved reward, the DDPG algorithm with improved experience pool, and the hybrid improved DDPG algorithm is 43.76, 61.34, 70.96, and 91.27%, respectively. Figure 13 gives a visual representation of the success rates of different DDPG algorithms. The DDPG algorithm with an improved reward and experience pool has higher average success rates than the original DDPG algorithm. Therefore, the improvement of the reward functions and the experience of the replay pool to the original DDPG algorithm is effective. The success rate of the hybrid reward function and experience replay pool improved by 47.51%. Therefore, the hybrid improved DDPG algorithm has better performance and stronger robustness.

**TABLE 1:** Comparison of robot arm arrival task success rates before and after deep deterministic policy gradient (DDPG) algorithm improvements.

Reward functions	Experience playback pool	Number of test rounds	Number of rounds completed	Success rate	Average success rate
Original	Original	1,000 2,000 5,000	522 875 1,766	52.20% 43.75% 35.32%	43.76%
Improvements	Original	1,000 2,000 5,000	574 1,307 3,064	57.40% 65.35% 61.28%	61.34%
Original	Improvements	2,000 1,000 5,000	1,481 707 3,407	74.05% 70.70% 68.14%	70.96%
Improvements	Improvements	2,000 5,000 1,000	**1,848** **4,516** **911**	**92.40%** **90.32%** **91.10%**	**91.27%**

Bold values indicate the experimental results for which the final improved algorithm is optimal.

**FIGURE 14:**
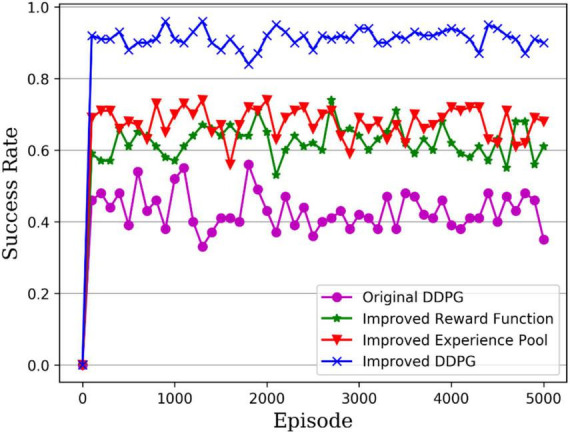
Test success rate of robotic arm arrival tasks.

Figure 14 presents the success rate curves for the different DDPG algorithms tested per 100 rounds, with the purple line being the original DDPG algorithm, the green line being the DDPG algorithm with improved reward, the red line being the DDPG algorithm with improved experience pool and the blue line being the hybrid improved DDPG algorithm. It can be seen that the success rate of the original DDPG algorithm fluctuates between 40 and 50%, while the success rates of both the DDPG algorithm with improved reward and with improved experience pools fluctuate between 60 and 70%, so the improvement of either the reward function or the experience pool increases the success rate of the robot arm in the reaching task. In contrast, the hybrid improved DDPG algorithm achieves a success rate of around 90%, which is much higher than the original DDPG algorithm and the other two improved DDPG algorithms, which shows more stability of the robotic arm arrival task control. Thus, the hybrid improved DDPG algorithm provides higher accuracy control of the robotic arm.

Combining the above experimental results, it can be seen that the improved DDPG algorithm proposed in this paper shows more robustness and higher learning efficiency compared to the original DDPG algorithm for the robotic arm-reaching task in an unknown working environment. In particular, the design of the hybrid reward function leads the robotic arm to the target object and provides a practical learning guide for the robotic arm. Compared to single-function rewards, it improves the exploration efficiency of reinforcement learning for the robotic arm. Moreover, the improvement in the experience replay mechanism, which can reuse historical sample data and filter out high-quality samples for network training, improves the convergence speed of the algorithm.

The improved DDPG algorithm proposed in this paper shows high learning efficiency and success rate in the 3D robotic arm reaching task. In addition, the experimental tasks performed in this paper are relatively single, and further verification of the generalization and robustness of the proposed algorithm requires doing more tasks in different unknown working environments, such as grasping robotic arms and other particular tasks with more difficulty.

## 5. Conclusion and future works

In response to the problem that traditional control methods of robotic arms in different environments still have poor self-adaptive capabilities, an intelligent control method of robotic arms based on the improved DDPG algorithm is proposed.

(1) This paper improves the reward function and experience replay mechanism in the original DDPG algorithm and proposes an improved DDPG algorithm suitable for robotic arms operating in dynamic environments.

(2) The simulation results show that both the reward function and the experience replay mechanism improve the success rate of the robotic arm in reaching the task. The average success rate of the improved DDPG algorithm is as high as 91.27%, and the convergence speed of the algorithm is accelerated.

The improved DDPG algorithm has a faster convergence speed and higher success rate, which gives the robotic arm the flexibility to choose the correct action and facilitates the efficient completion of a specific task. For future work, the improved method proposed in this paper can be tried to be applied in a physical robotic arm. Explore the method’s effectiveness in robotic arm grasping and combine it with vision algorithms to accomplish more complex control decision tasks.

## Data availability statement

The original contributions presented in this study are included in this article/supplementary material, further inquiries can be directed to the corresponding author.

## Author contributions

RD, JD, YL, and AH: writing—original draft, writing—review and editing, software, visualization, and investigation. HC: conceptualization, methodology, formal analysis, investigation, writing—review and editing, funding acquisition, and supervision. All authors contributed to the article and approved the submitted version.
